# Identification of Suitable Reference Genes for Real-Time PCR Analysis of Statin-Treated Human Umbilical Vein Endothelial Cells

**DOI:** 10.1371/journal.pone.0051547

**Published:** 2012-12-10

**Authors:** Barbara Żyżyńska-Granica, Katarzyna Koziak

**Affiliations:** Department of General and Nutritional Biochemistry, Medical University of Warsaw, Warsaw, Poland; Morehouse School of Medicine, United States of America

## Abstract

Proper data normalization in quantitative real-time reverse-transcription polymerase chain reaction (RT-qPCR) is of critical importance for reliable mRNA expression analysis. Due to a diversity in putative reference genes expression stability in different *in vitro* models, a validation of an internal control gene should be made for each particular tissue or cell type and every specific experimental design. A few approaches have been proposed for reference gene selection, including pair-wise comparison approach and model-based approach. In this article we have assessed the expression stability of eight putative reference genes: *ACTB*, *B2M*, *GADD45A*, *GAPDH*, *HPRT1*, *PES1*, *PSMC4*, *YWHAZ*, in human umbilical vein endothelial cells (HUVEC) treated with different statins and with TNF-α. The analysis was performed with three reference gene validation programs: *geNorm*, *NormFinder* and *BestKeeper*. We have shown that hypoxanthine phosphoribosyltransferase 1 gene (*HPRT1*) and tyrosine 3-monooxygenase/tryptophan 5-monooxygenase activation protein, zeta polypeptide gene (*YWHAZ*) are the most stably expressed genes among the analyzed ones. Furthermore, our results show that β-actin gene (*ACTB*) is downregulated by statins and thus should not be used as a normalizing gene in a discussed experimental setup. A ranking of candidate reference genes stability values is provided and might serve as a valuable guide for future gene expression studies in endothelial cells. This is the first report on reference gene selection for RT-qPCR applications in statin-treated HUVEC model.

## Introduction

Quantitative real-time reverse-transcription polymerase chain reaction (RT-qPCR) has become one of the most popular techniques of quantifying mRNA levels. The method is relatively easy and precise, however, when used in an inappropriate way, it can lead to considerable misinterpretation of results [1]–[4]. It is therefore of critical importance to perform proper data normalization which enables to control differences between samples that may arise at different stages throughout the procedure. However, although the qPCR has become very popular, data normalization still remains a problem. There are several strategies which can be applied to normalize qPCR results [3], but the most common one is the use of reference genes as an internal standard. Although recent studies clearly show the importance of a proper choice of a reference gene [2], [5], [6], still many currently published reports present RT-qPCR results that miss information on a reference gene selection. In addition, researchers often routinely use the most classical reference genes, such as genes coding for glyceraldehyde 3-phosphate dehydrogenase (*GAPDH*) or β-actin (*ACTB*), convinced that these are the universal reference genes and unaware that they can be highly regulated [7]–[9].

The ideal reference genes should be expressed at the same level in all cells and under all experimental conditions. It has been, however, well documented, that most of them undergo significant regulation and thus cannot be considered as a proper reference [10]. Despite these limitations, the use of reference genes as internal controls remains the most common method used to normalize cellular mRNA content in analyzed samples [11], [12]. A fortiori, the use of this method should be preceded with rigorous reference genes validation to avoid an improper gene choice. Therefore, for each particular tissue or cell type and specific experimental designs, a thorough search is needed to ensure that no significant change in a reference gene expression occurs [13]. Unfortunately, despite its importance, this experimental step is often neglected.

The aim of this study was to identify the most stable reference genes for human umbilical vein endothelial cells (HUVEC) treated with different 3-hydroxy-3-methylglutaryl-coenzyme A (HMG-CoA) reductase inhibitors, known as statins. HMG-CoA reductase inhibitors are among the most frequently prescribed drugs for the prevention and treatment of cardiovascular diseases. Besides lowering the plasma cholesterol concentration, they exert pleiotropic effects that are independent of their cholesterol-lowering properties [14], [15], which include improved endothelial functions and decreased vascular inflammation [14]. Although HUVEC are often used as an *in vitro* model to determine mechanisms of statins effects on endothelial cells and expression of various genes is analyzed using RT-qPCR method, very little is known about the stability of potential reference genes used in such studies [16]. We have assessed the expression stability of eight putative reference genes in HUVEC treated with six different statins: lovastatin, atorvastatin, fluvastatin, simvastatin, pravastatin and cerivastatin and additionally stimulated with a proinflammatory cytokine, tumor necrosis factor alpha (TNF-α). The reference genes examined were: β-actin (*ACTB*), glyceraldehyde 3-phosphate dehydrogenase (*GAPDH*), β-2-microglobulin (*B2M*), growth arrest and DNA-damage-inducible protein alpha (*GADD45A*), hypoxanthine phosphoribosyltransferase 1 (*HPRT1*), pescadillo homolog 1 containing BRCT domain (zebrafish) (*PES1*), proteasome (prosome, macropain) 26S subunit, ATPase 4 (*PSMC4*) and tyrosine 3-monooxygenase/tryptophan 5-monooxygenase activation protein, zeta polypeptide (*YWHAZ*). We believe that the presented data related to a reference gene selection will be useful not only for the RT-qPCR analyses of statin-treated HUVEC, but also for other studies with human primary cells.

## Materials and Methods

### Experimental Design

In the present study eight candidate reference genes (*ACTB*, *B2M*, *GADD45A*, *GAPDH*, *HPRT1*, *PES1*, *PSMC4*, *YWHAZ*) (Table 1) were evaluated in HUVEC which underwent statin or combined statin and TNF-α treatments. All the necessary controls were included. All HMG-CoA reductase inhibitors, i.e. lovastatin, atorvastatin, fluvastatin, simvastatin, pravastatin and cerivastatin, were used at concentrations which did not induce any cytotoxicity (data not shown). All the selected candidate reference genes belong to different functional classes to minimize the chance of their co-regulation.

**Table 1:** Putative reference genes evaluated.

Symbol	Gene name	Function
*ACTB*	β-actin	Cytoskeletal structural protein
*B2M*	β-2-microglobulin	Beta-chain of major histocompatibility complex class I molecules
*GADD45A*	growth arrest and DNA-damage-inducible protein, alpha	Cell cycle regulation in stressful conditions
*GAPDH*	Glyceraldehyde-3-phosphate dehydrogenase	Oxidoreductase in glycolysis and gluconeogenesis
*HPRT1*	hypoxanthine phosphoribosyltransferase 1	Central role in the purine metabolism through the purine salvage pathway
*PES1*	pescadillo homolog 1, containing BRCT domain (zebrafish)	Cell proliferation
*PSMC4*	proteasome (prosome, macropain) 26S subunit, ATPase, 4	Protein ubiquitination, ATP catabolism
*YWHAZ*	tyrosine 3-monooxygenase/tryptophan 5-monooxygenaseactivation protein, zeta polypeptide	Signal transduction by binding to phosphorylated serine residues on a variety of signaling molecules

Data were analyzed in three reference gene validation programs: *geNorm* [17], *NormFinder* [18] and *BestKeeper* [13], and the results were used to rank the candidate reference genes from the most to least stable. Based on the rankings obtained from each program, which assigned appropriate weights to every individual gene, the geometric mean of their weights was calculated for the overall final rankings. Candidate reference genes were analyzed thrice using samples from three different cell donors. 20 cDNAs containing statin and statin-and-TNF-α samples were obtained from each donor. The obtained data were analyzed for each donor either separately for statin-treated cells and statin-and-TNF-α-treated cells or in the pooled analysis of all 20 samples.

### Cell Culture and Treatment

HUVEC were purchased from Invitrogen Life Technologies. Three populations from different cell donors were used. Cells were grown in EBM-2 basal medium supplemented with the EGM-2 SingleQuots kit (Lonza, USA). For all experiments HUVEC at passage four were used.

Cells were grown to confluence and then treated with lovastatin at final concentrations 1 µM and 2 µM, atorvastatin 1 µM and 2 µM, fluvastatin 1 µM and 2 µM, simvastatin 1 µM, cerivastatin 0.1 µM or pravastatin 1 mM for 48 hours in full EGM-2 medium. Untreated cells were used as controls. The same experimental setup was repeated and followed with TNF-α treatment (10 ng/mL, 1 hour).

### RNA Isolation and cDNA Synthesis

Total RNA was extracted from HUVEC and purified using the NucleoSpin RNA II Kit (Marcherey-Nagel, Germany) according to the manufacturer’s instruction. Cell lysis was performed in a RNases inactivating buffer provided by the manufacturer. Until RNA extraction samples were stored at −70°C. Purified RNA was reverse transcribed immediately after extraction.

RNA concentrations and 260/280 absorbance ratios were measured spectrophotometrically with an Ultrospec 3000 (Pharmacia Biotech, UK).

cDNA was synthesized using a High Capacity cDNA Reverse Transcription Kit (Applied Biosystems, USA) following manufacturer’s instructions. The reaction was set with 6 µg of total RNA in a total volume of 60 µL containing: random primers, 4 mM deoxyribonucleotide triphosphates (dNTP’s), 2.5 U/µL MultiScribe Reverse Transcriptase and RT buffer. RNase inhibitor (1 U/µL) was used for each reverse transcription PCR reaction. Cycle parameters were set to 10 minutes at 25°C, 120 minutes at 37°C and 5 minutes at 85°C. cDNA was stored at −20°C until further use.

### Quantitative Real-time Reverse-transcription PCR

The following eight putative reference genes were selected for analysis: *ACTB*, *B2M*, *GADD45A*, *GAPDH*, *HPRT1*, *PES1*, *PSMC4* and *YWHAZ*. The selected genes belong to different functional classes, which reduces the chance of co-regulation.

All primers and probes were purchased from Applied Biosystems, USA (Table 2). The real-time qPCR reactions were performed using TaqMan® Gene Expression Assays (FAM™ dye-labeled MGB probes) and TaqMan® Gene Expression Master Mix (2X) (PN 4369016, Applied Biosystems, USA) exactly to the manufacturer’s instructions.

**Table 2:** Details of primers for evaluated genes and RT-qPCR amplification efficiencies.

Symbol	Cat No	Amplicon size	UniGene No	Gene Bank Accession No	Efficiency
*ACTB*	Hs99999903_m1	171	Hs.520640	NM_001101.3	101.1%
*B2M*	Hs99999907_m1	75	Hs.534255	NM_004048.2	96.2%
*GADD45A*	Hs00169255_m1	123	Hs.80409	NM_001199741.1	99.8%
*GAPDH*	Hs99999905_m1	122	Hs.479728	NM_002046.3	99.1%
*HPRT1*	Hs99999909_m1	100	Hs.412707	NM_000194.2	96.1%
*PES1*	Hs00362795_g1	56	Hs.517543	NM_001243225	94.7%
*PSMC4*	Hs00197826_m1	83	Hs.211594	NM_006503.2	90.5%
*YWHAZ*	Hs00237047_m1	70	Hs.492407	NM_003406.3	90.7%

The real-time qPCR reactions were performed on the 7500 Real-Time PCR System (Applied Biosystems, USA) in MicroAmp® Optical 96-Well Reaction Plates (PN 4306737, Applied Biosystems, USA) manually set up in triplicates.

PCR conditions were as follows: 50°C for 2 minutes, 95°C for 10 minutes followed by 40 cycles of 95°C for 15 seconds (denaturation step) and 60°C for 60 seconds (annealing and extension step) during which fluorescence was measured. Data expression levels were recorded as quantification cycles (C_q_). Data was acquired using the 7500 Software (Applied Biosystems, USA). The mean C_q_ values of the triplicate reactions were used in further analysis.

Calculations of the mean C_q_ and fold change values were performed by means of DataAssist v3.0 Software (Applied Biosystems, USA).

### PCR Efficiency

A 2-fold dilution series was prepared from pooled cDNA samples. PCR reactions were performed as described above in triplicates. The PCR efficiencies (*E*) and correlation coefficients (*R^2^*) for each primer pair were calculated using the formula: *E (%) = (10^(-1/slope)^ - 1)•100*. Slopes were determined from a standard curve obtained when a logarithm of the initial template concentration was plotted on the *x* axis and C_q_ on the *y* axis [11]. The efficiencies for all primer pairs are listed in Table 2.

### Data Analysis

#### GeNorm analysis

*GeNorm* is a software for Microsoft Excel which provides a measure of gene expression stability [17]. It ranks the genes basing on the internal control gene stability parameter *M*. *M* is the mean pair-wise variation between individual gene and the other putative reference genes tested. Stepwise exclusion of the gene with the highest *M* value and recalculation allows ranking of the tested genes according to their expression stability. Lower *M* values represent higher expression stabilities. Any gene with *M* >1.5 is suggested to be considered unreliable as a stable reference gene [17]. *GeNorm* authors suggest the use of 10 samples and 8 reference genes for validation procedure.

#### Normfinder analysis

*NormFinder* is another Excel-based statistical algorithm that computes expression stability values to range candidate reference genes [18]. A high stability value represents a high gene expression variance. In addition, the program allows for comparison of inter- and intra-group variation of gene stability. *NormFinder* authors suggest using at least 8 samples per group and minimum 3 candidate genes, but recommend 5–10 genes [18].

#### BestKeeper analysis

*BestKeeper* analyses C_q_ values to evaluate the expression variability of the reference genes. The key factor in the analysis is the standard deviation (SD) which represents the stability of the gene, and the lower SD value, the better stability. Any studied gene with the SD >1 is suggested to be considered unreliable [13].

Afterwards, the program performs a comparative analysis based on pair-wise correlation coefficient (*r*) between each gene and the *BestKeeper* Index (BI), which is the geometric mean of C_q_ values of candidate reference genes.

The genes with SD >1 are eliminated from further analysis and the remaining genes are ranked according to their coefficient of correlation (*r*).

#### Final ranking

All analyzed genes were ranked by all three programs and appropriate weights to every gene were assigned. For the overall final rankings the geometric means of the obtained weights were calculated.

## Results

For each primer pair PCR efficiencies were calculated from the slope of the standard curve. The obtained efficiencies varied from 90.5% to 101.1% (Table 2).

### First Donor Assay

The first assay was performed with a set of eight primer pairs (*ACTB*, *B2M*, *GADD45A*, *GAPDH*, *HPRT1*, *PES1*, *PSMC4*, *YWHAZ*) for 20 cDNA samples, i.e. statin and combined statin-and-TNF-α-treated cells from the first donor. C_q_ values were used for further analysis. Three reference gene validation programs were used: *geNorm*, *NormFinder* and *BestKeeper*. Mean C_q_ values were input into *BestKeeper*. For *geNorm* and *NormFinder* C_q_ values were transformed into relative quantification data using the equation 2^(-ΔCq)^. ΔC_q_ is the difference between data point of interest and the highest data point in the data set. Therefore all data is relative to the sample showing the lowest level of gene expression. For pooled analysis of 20 cDNA samples, including statin- and combined statin-and-TNF-α-treated samples, *GeNorm* ranked analyzed genes basing on their stability value (*M*). The most stably expressed genes, with the lowest *M*-value, were *HPRT1* and *YWHAZ* (Table 3, part A). *NormFinder* also ranges genes depending on a stability value but using different algorithm. The lowest stability value was for *B2M* and the second lowest value was for *GAPDH* (Table 3, part A). *BestKeeper* produced descriptive statistics (data not shown). For all the analyzed genes, except for *ACTB*, SD values were below 1 (Table 3, part A), which suggests that *ACTB* should be excluded from further analysis and the rest of analyzed genes could be potentially used as reference genes. The ranking of putative reference genes based on coefficient of correlation values (*r*) is shown in Table 3, part A. The best correlated genes were *YWHAZ* followed by *HPRT1*. Next, geometric means of the weights from all three rankings for every individual gene were calculated and are presented in Table 3, part A. In the final ranking *YWHAZ* is positioned first and is followed by *HPRT1*.

**Table 3:** Overall comparison of putative reference genes’ stability.

Rank (weight)	Program																	
	*geNorm*				*NormFinder*			*BestKeeper*						*Final ranking*				
	*Gene*		*M-value*		*Gene*		*Stability value*	*Gene*		*R*		*SD*		*Gene*		*GeoMean*		
**A. First donor assay**																		
**1**		HPRT1		0.245		B2M	0.178		YWHAZ		0.993		0.58		**YWHAZ**		**1.44**	
**2**		YWHAZ		0.245		GAPDH	0.252		HPRT1		0.982		0.69		**HPRT1**		**2.00**	
**3**		B2M		0.397		YWHAZ	0.377		B2M		0.961		0.64		**B2M**		**2.08**	
**4**		GAPDH		0.430		HPRT1	0.380		GAPDH		0.878		0.95		**GAPDH**		**3.17**	
**5**		PSMC4		0.543		ACTB	0.384		PSMC4		0.865		0.96		**PSMC4**		**5.59**	
**6**		ACTB		1.041		PES1	0.447		PES1		0.864		0.57		**ACTB**		**6.21**	
**7**		PES1		1.048		PSMC4	0.594		GADD45A		0.783		0.51		**PES1**		**6.32**	
**8**		GADD45A		1.856		GADD45A	1.164		ACTB		-		1.08		**GADD45A**		**7.65**	
**B. Second donor assay**																		
**1**		HPRT1		0.185		B2M	0.089		YWHAZ		0.980		0.45		**YWHAZ**		**1.26**	
**2**		YWHAZ		0.185		YWHAZ	0.279		PSMC4		0.973		0.51		**HPRT1**		**2.29**	
**3**		PSMC4		0.257		GAPDH	0.283		HPRT1		0.949		0.62		**B2M**		**2.71**	
**4**		GAPDH		0.280		HPRT1	0.295		B2M		0.946		0.49		**PSMC4**		**3.48**	
**5**		B2M		0.456		PES1	0.369		PES1		0.926		0.35		**GAPDH**		**4.38**	
**6**		ACTB		0.760		ACTB	0.376		ACTB		0.856		0.79		**PES1**		**5.59**	
**7**		PES1		0.871		PSMC4	0.420		GAPDH		0.813		0.59		**ACTB**		**6.00**	
**8**		GADD45A		1.833		GADD45A	1.130		GADD45A		0.777		0.47		**GADD45A**		**8.00**	
**C. Third donor assay**																		
**1**		YWHAZ		0.213		PSMC4	0.091		PSMC4		0.923		0.34		**PSMC4**		**1.59**	
**2**		B2M		0.213		HPRT1	0.137		HPRT1		0.890		0.37		**HPRT1**		**2.29**	
**3**		HPRT1		0.313		PES1	0.151		PES1		0.878		0.42		**B2M**		**2.52**	
**4**		PSMC4		0.357		B2M	0.163		B2M		0.856		0.44		**YWHAZ**		**2.92**	
**5**		PES1		0.378		YWHAZ	0.198		YWHAZ		0.840		0.37		**PES1**		**3.56**	
**6**		GAPDH		0.477		ACTB	0.199		GAPDH		0.839		0.68		**GAPDH**		**6.32**	
**7**		ACTB		0.578		GAPDH	0.361		GADD45A		0.816		0.33		**ACTB**		**6.95**	
**8**		GADD45A		0.934		GADD45A	0.568		ACTB		0.809		0.55		**GADD45A**		**7.65**	

Results are calculated for all samples for each donor, i.e. statin-treated cells and combined statin-and-TNF-α-treated cells. Rankings are based on *geNorm* stability M-values, *NormFinder* stability values and coefficient of correlation values (r) counted by *BestKeeper*. SD values calculated by *BestKeeper* are also given in the table. The genes with SD >1 are eliminated from further analysis. For the overall final ranking the geometric mean of the weights (*GeoMean*) assigned by the rankings from all three programs was calculated.

The statin- and combined statin-and-TNF-α-treated samples were also analyzed separately. For statin-treated samples the most stably expressed genes according to *geNorm* analysis were *HPRT1* and *B2M*. *NormFinder* ranked *HPRT1* at the first and *YWHAZ* at the second position. According to *BestKeeper* the best correlated genes were *YWHAZ* and *HPRT1*. In the final ranking *HPRT1* was positioned first and *YWHAZ* second. The results are presented in Table 4, part A.

**Table 4:** Overall comparison of putative reference genes’ stability for statin-treated cells.

Rank (weight)	Program																		
	*geNorm*				*NormFinder*				*BestKeeper*						*Final ranking*				
	*Gene*		*M-value*		*Gene*		*Stability value*		*Gene*		*R*		*SD*		*Gene*		*GeoMean*		
**A. First donor assay**																			
**1**		HPRT1		0.168		HPRT1		0.058		YWHAZ		0.998		0.74		**HPRT1**		**1.26**	
**2**		B2M		0.168		YWHAZ		0.068		HPRT1		0.993		0.87		**YWHAZ**		**2.00**	
**3**		PES1		0.195		PSMC4		0.086		PSMC4		0.991		0.76		**B2M**		**2.92**	
**4**		YWHAZ		0.283		PES1		0.087		PES1		0.986		0.90		**PSMC4**		**3.56**	
**5**		PSMC4		0.306		B2M		0.147		B2M		0.979		0.89		**PES1**		**3.63**	
**6**		GAPDH		0.511		GAPDH		0.317		GAPDH		0.933		0.93		**GAPDH**		**6.00**	
**7**		GADD45A		0.541		GADD45A		0.387		GADD45A		0.891		0.74		**GADD45A**		**7.00**	
**8**		ACTB		1.046		ACTB		0.697		ACTB		-		1.40		**ACTB**		**8.00**	
**B. Second donor assay**																			
**1**		HPRT1		0.145		YWHAZ		0.050		YWHAZ		0.990		0.58		**YWHAZ**		**1.00**	
**2**		YWHAZ		0.145		PSMC4		0.052		HPRT1		0.987		0.67		**HPRT1**		**1.82**	
**3**		PSMC4		0.192		HPRT1		0.063		PSMC4		0.986		0.58		**PSMC4**		**2.62**	
**4**		B2M		0.261		GAPDH		0.129		GAPDH		0.964		0.64		**GAPDH**		**4.31**	
**5**		GAPDH		0.263		PES1		0.166		PES1		0.954		0.44		**B2M**		**5.24**	
**6**		PES1		0.289		B2M		0.170		B2M		0.946		0.66		**PES1**		**5.31**	
**7**		GADD45A		0.337		GADD45A		0.255		GADD45A		0.869		0.47		**GADD45A**		**7.00**	
**8**		ACTB		0.902		ACTB		0.614		ACTB		0.805		0.95		**ACTB**		**8.00**	
**C. Third donor assay**																			
**1**		YWHAZ		0.099		HPRT1		0.042		HPRT1		0.988		0.38		**HPRT1**		**1.44**	
**2**		PSMC4		0.099		B2M		0.043		B2M		0.986		0.43		**YWHAZ**		**2.08**	
**3**		HPRT1		0.158		YWHAZ		0.045		YWHAZ		0.985		0.35		**B2M**		**2.71**	
**4**		GAPDH		0.176		GAPDH		0.077		PSMC4		0.966		0.30		**PSMC4**		**2.71**	
**5**		B2M		0.181		PSMC4		0.101		GAPDH		0.958		0.39		**GAPDH**		**4.31**	
**6**		PES1		0.202		PES1		0.117		PES1		0.933		0.42		**PES1**		**6.00**	
**7**		GADD45A		0.341		GADD45A		0.256		GADD45A		0.869		0.55		**GADD45A**		**7.00**	
**8**		ACTB		0.565		ACTB		0.380		ACTB		0.751		0.54		**ACTB**		**8.00**	

Results are calculated for 10 statin-treated samples for each donor. Rankings are based on *geNorm* stability M-values, *NormFinder* stability values and coefficient of correlation values (r) counted by *BestKeeper*. SD values calculated by *BestKeeper* are also given in the table. For the overall final ranking the geometric mean of the weights (*GeoMean*) assigned by the rankings from all three programs was calculated.

For combined statin-and-TNF-α-treated samples *geNorm* analysis indicated *HPRT1* and *B2M* as the best reference genes. *NormFinder* ranked *YWHAZ* as the best reference gene followed by *PSMC4*. The best correlated genes according to *BestKeeper* were *HPRT1* followed by *GAPDH*. Final ranking indicated *HPRT1* as the best reference gene and *YWHAZ* as the second best reference gene (data not shown).

### Second Donor Assay

The second donor assay was also performed with 20 cDNA samples and the eight primer sets evaluated in this study. The methods of analysis were the same as previously. For pooled analysis of 20 cDNA samples, including statin- and combined statin-and-TNF-α-treated samples, the most stably expressed genes according to *geNorm* are *HPRT1* and *YWHAZ*. *NormFinder* indicated *B2M* and *YWHAZ* as the best and the second best reference genes respectively. *BestKeeper* analysis ranked *YWHAZ* as the best reference gene followed by *PSMC4*. In the final ranking, similarly to the first cell donor, *YWHAZ* and *HPRT1* were ranked as the best and the second best reference genes respectively. The results are presented in Table 3, part B.

Again, samples obtained from statin-treated cells were analyzed separately from combined statin-and-TNF-α-treated samples. All respective controls were included in the analyses. The most stably expressed genes for statin-treated cells according to *geNorm* analysis were *HPRT1* and *YWHAZ*. *NormFinder* analysis suggested *YWHAZ* as the best and *PSMC4* as the second best reference genes. *HPRT1* was ranked at the third position. *BestKeeper* analysis ranked *YWHAZ* at the first and *HPRT1* at the second position. For all analyzed genes SD values were below 1. Final ranking ranked *YWHAZ* at the first position followed by *HPRT1*. The results are presented in Table 4, part B.

For samples treated with statins and TNF-α *geNorm* ranked *B2M* and *YWHAZ* as the two best reference genes. *NormFinder* positioned *PSMC4* and *YWHAZ* at the first and second position respectively. According to *BestKeeper* analysis the best reference gene was *HPRT1* followed by *B2M*. In the final ranking *B2M* and *PSMC4* were ranked at the first two positions. *YWHAZ* and *HPRT1* were ranked at the third and fourth position respectively (data not shown).

### Third Donor Assay

Similarly to the first and second donor, the third donor assay was performed with eight primer sets and 20 cDNA. The methods of analysis were the same as previously. For pooled analysis of 20 cDNA samples, including statin- and combined statin-and-TNF-α-treated samples, the best reference genes indicated by *geNorm* analysis were *B2M* and *YWHAZ*. *NormFinder* ranked *PSMC4* at the first and *HPRT1* at the second position. According to *BestKeeper* the best correlated reference genes were *PSMC4* and *HPRT1*. In the final ranking *PSMC4* was ranked first and *HPRT1* second. *YWHAZ* was positioned fourth. The results are presented in Table 3, part C.

In a manner analogous to the first and the second donor assays, results obtained for statin-treated samples were analyzed separately from combined statin-and-TNF-α-treated samples. All respective controls were included in the analyses. *GeNorm* analysis indicated *YWHAZ* and *PSMC4* as the two best reference genes for RT-qPCR studies with statin-treated HUVEC. *NormFinder* and *BestKeeper* ranked *HPRT1* as the best and *B2M* as the second best reference gene. In final ranking *HPRT1* was ranked first and *YWHAZ* second. The results are presented in Table 4, part C.

For combined statin-and-TNF-α-treated samples *geNorm* ranked *HPRT1* and *GAPDH* as the two best reference genes. According to *NormFinder* and *BestKeeper GAPDH* and *PSMC4* were the best and second best reference genes respectively. At the final ranking *GAPDH* was positioned highest and was followed by *HPRT1*. *YWHAZ* was ranked fourth (data not shown).

## Discussion

RT-qPCR has become a gold standard for quantifying mRNA. As this method of analyzing gene expression is highly specific and relatively easy, it has reached a great popularity. However, the data normalization still remains an issue. The most common method of normalizing qPCR results is the use of reference genes and the strategy is based on the assumption that they are stably expressed. However, many studies have demonstrated that the stability of each reference gene needs to be verified individually under specific experimental conditions [7]–[9]. It is also recommended that a series of genes are tested for stability and more than one is used for normalization in the final experimental setup. When a gene of interest is not compared to appropriately validated, stably expressed reference genes, misinterpretation of results may occur. Constantly growing evidence indicates that there is no single reference gene that can be used for different experiments, but hopefully with the growing number of experimental data and reports, such as this one, a group of putative reference genes for certain specific experimental setups could be recommended for future studies [5], [6], [19], [20].

In this study we have shown the variability in the expression stability of eight putative reference genes (*ACTB*, *B2M*, *GADD45A*, *GAPDH*, *HPRT1*, *PES1*, *PSMC4*, *YWHAZ*) in statin-treated HUVEC when compared in three Excel-based programs: *geNorm*, *NormFinder* and *BestKeeper*. As for the validation of a reference gene only limited number of samples from all to be analyzed are usually used, we decided to verify the impact of the samples’ selection on the obtained results. For this purpose we performed three assays with three sample sets collected from different cell donors. We have also verified weather the reference genes selected for statin-treated cells may also be used for RT-qPCR analysis of cells additionally stimulated with TNF-α. Therefore, for each cell donor three sets of analyses were performed: 1) for statin-treated cells, 2) for combined statin-and-TNF-α-treated cells, and 3) pooled analysis for all the samples.

Although the results obtained for every donor differ to some extent, certain putative reference genes (i.e. *HPRT1*, *YWHAZ* and *B2M*) are ranked high in most of the analyses, while the other (i.e. *ACTB*, *GADD45A* and *PES1*) are predominantly ranked low.

In all the analyses of statin-treated cells, *ACTB*, one of the most popular reference genes, has been ranked at the last position (Table 4) suggesting that statins affect its expression. A more detailed evaluation of the obtained results has indicated that the expression of *ACTB* is down-regulated by all statins (data not shown), what excludes it from a group of potential reference genes for the presented type of experiments.

*GAPDH*, another reference gene often used to normalize RT-qPCR data without any validation, has been ranked low indicating that it is not suitable for this research model. These results show that the validation of a reference gene for normalizing RT-qPCR data is crucial and using popular reference genes, such as *ACTB* or *GAPDH*, without any validation may lead to false results.

Summarizing, *HPRT1* and *YWHAZ* were ranked high in all the analyses which makes these genes the best choice for normalizing gene expression in statin-treated HUVEC. As it is commonly suggested to use more than one reference gene for normalizing data in qPCR studies [3], we recommend these two genes as reference genes in the presented experimental setup.

The differences in the results obtained from the first, second and third donor assays may reflect a normal genetic diversity of human population. HUVEC are primary cells isolated from human umbilical vein and in this study each pool has originated from a different donor. This might be the reason for some differences in the genes regulation in the presented experimental setup leading to the disparity of the rankings. Therefore, our study shows that the validation of reference genes for experiments based on heterogeneous cell cultures requires more samples than recommended by *geNorm* and *NormFinder* authors.

The disparities between the programs output in each analysis are a result of different methodologies used in the calculation of gene stabilities (e.g. model-based approach and pair-wise variation evaluation) and overall inherent variability of the genes examined. The pair-wise comparison approach (*geNorm*, *BestKeeper*) selects the most suitable reference gene on the basis of the variation of expression ratios between candidate reference genes expression across the sample set. It is based on the assumption that the ratio between two putative reference genes is constant across samples independently to RNA amount analyzed per sample. The variation of this ratio for two candidate reference genes across samples (pair-wise variation) is a measure of gene stability. However, *geNorm* and *BestKeeper* algorithm analyses are based on the assumption that none of the genes analyzed in the study is co-regulated.

The co-regulation of candidate reference genes does not significantly affect the model-based approach (*NormFinder*). Nevertheless, this type of analysis can be sensitive to sampling errors and outliners. For that reason the use of more than one type of algorithm for the validation of reference genes is suggested. The comparison of the reference genes rankings obtained from more than one program will give more reliable results.

This report should draw particular attention to a proper experimental planning. In the optimal setup reference gene validation should be carried out for every experiment and every pool of samples, but as the process is time and money consuming this recommendation seems difficult to achieve. Nevertheless, our study clearly shows that the more samples used for the validation of the reference gene the better. The same rule applies to choosing reference genes, however, the possibility that some of the selected genes are co-regulated and thus may falsify the results should be considered.

In conclusion, in this study we have shown that among the analyzed genes, *HPRT1* and *YWHAZ* are the most suitable reference genes for the expression studies in HUVEC treated with statins and additionally stimulated with TNF-α. Moreover, our results clearly show that *ACTB* should not be used as a normalizing gene in a discussed experimental setup. These data may also be useful when validating reference genes for other studies with HUVEC. Our observations confirm that the proper selection of a reference gene is crucial for reliable data analyzing.
